# Methodology for a mixed-methods multi-country study to assess recognition of and response to maternal and newborn illness

**DOI:** 10.1186/s41043-017-0119-8

**Published:** 2017-12-21

**Authors:** Allisyn C. Moran, Danielle Charlet, Supriya Madhavan, Kumudha Aruldas, Marie Donaldson, Fatuma Manzi, Monica Okuga, Alfonso Rosales, Vandana Sharma, Michael Celone, Neal Brandes, James M. Sherry

**Affiliations:** 1 United States Agency for International Development, Bureau for Global Health, Washington, DC, USA; 2 University Research Co., LLC, Bethesda, MD USA; 3 Population Council, New Delhi, India; 4 Ifakara Health Institute, Dar es Salaam, Tanzania; 5 Makerere University School of Public Health, Makerere University College of Health Sciences, Kampala, Uganda; 6 World Vision, Washington, DC, USA; 7 The Abdul Latif Jameel Poverty Action Lab, Massachusetts Institute of Technology, Cambridge, MA USA; 8 School of Public Health, City University of New York, New York, New York USA

**Keywords:** Maternal mortality, Newborn mortality, Developing country, Qualitative research, Care-seeking behavior

## Abstract

**Background:**

Although maternal and newborn mortality have decreased 44 and 46% respectively between 1990 and 2015, achievement of ambitious Sustainable Development Goal targets requires accelerated progress. Mortality reduction requires a renewed focus on the continuum of maternal and newborn care from the household to the health facility. Although barriers to accessing skilled care are documented for specific contexts, there is a lack of systematic evidence on how women and families identify maternal and newborn illness and make decisions and subsequent care-seeking patterns. The focus of this multi-country study was to identify and describe illness recognition, decision-making, and care-seeking patterns across various contexts among women and newborns who survived and died to ultimately inform programmatic priorities moving forward.

**Methods:**

This study was conducted in seven countries—Ethiopia, Tanzania, Uganda, Nigeria, India, Indonesia, and Nepal. Mixed-methods were utilized including event narratives (group interviews), in-depth interviews (IDIs), focus group discussions (FDGs), rapid facility assessments, and secondary analyses of existing program data. A common protocol and tools were developed in collaboration with study teams and adapted for each site, as needed. Sample size was a minimum of five cases of each type (e.g., perceived postpartum hemorrhage, maternal death, newborn illness, and newborn death) for each study site, with a total of 84 perceived PPH, 45 maternal deaths, 83 newborn illness, 55 newborn deaths, 64 IDIs/FGDs, and 99 health facility assessments across all sites. Analysis included coding within and across cases, identifying broad themes on recognition of illness, decision-making, and patterns of care seeking, and corresponding contextual factors. Technical support was provided throughout the process for capacity building, quality assurance, and consistency across sites.

**Conclusion:**

This study provides rigorous evidence on how women and families recognize and respond to maternal and newborn illness. By using a common methodology and tools, findings not only were site-specific but also allow for comparison across contexts.

## Background

Significant progress has been made toward achieving the fourth and fifth United Nations Millennium Development Goals (MDGs) to reduce child and maternal mortality. Between 1990 and 2015, neonatal mortality declined by 47% and currently comprises almost half of under-five deaths worldwide [1]. Maternal mortality declined by 44%, far less than the three-quarters reduction target [2]. To continue to address maternal, newborn, and child mortality, the Sustainable Development Agenda includes ambitious global targets to be attained by 2030 [3].

Achieving these global targets will require a renewed focus on effective coverage inclusive of quality and respectful maternal and newborn care. There are a variety of factors that inhibit timely and appropriate care seeking for maternal and newborn complications. For both women and newborns, improving the availability of care will not effectively reduce maternal and neonatal mortality unless families know when, where, and how to seek it. In 1994, Thaddeus and Maine developed the Three Delays model which organizes barriers to identification and care seeking for maternal complications, including (1) delay in deciding to seek care; (2) delay in reaching a facility; and (3) delay in receiving quality care [4]. The MotherCare project adapted the Three Delays model by dividing the first delay into two steps: (1) recognition of the problems and (2) decision-making [5]. This framework has also been successfully applied to care seeking for newborn illness [6].

The Three Delays model and MotherCare project framework haves been used to both develop and evaluate maternal/newborn programs and have been adapted by a variety of programs over time. The aim of this multi-country study was to explore the first two delays across different contexts to systematically document how women and families identify maternal and newborn illness, the factors that influence decision-making, and care-seeking patterns. Better understanding of the drivers behind these constructs can inform development of more effective programs and policies to ultimately improve maternal and newborn survival. The objective of this paper is to provide an overview of the common methodology for this study.

### Review of literature

A structured literature review was conducted to document evidence on the first two delays. Search terms were entered into the EBSCOhost and PubMed databases, including key words maternal health, newborn health, care seeking, recognition, and danger signs. Abstracts were reviewed for relevance based on the inclusion criteria: (1) papers published after 1975; (2) studies in low- and middle-income countries; and (3) focus on recognition and care seeking for maternal or neonatal complications.

### Delay 1: illness recognition

#### Maternal

The literature identified several signs and symptoms of maternal illness as well recognized among women and caretakers, including vaginal bleeding [7–11]. Several factors are associated with knowledge of these signs and symptoms among women, families, and caregivers, including educational status, having a radio, place of delivery [9], higher age, parity, number of antenatal care visits, delivery location [12], religion, ethnicity, and professional occupation [11].

There are several signs/symptoms that present along a continuum, at one end of which they are natural or physiological and only become potentially life-threatening as severity increases, such as prolonged labor or postpartum hemorrhage. It can be difficult for family members to recognize the point at which a normal sign/symptom becomes life-threatening. For instance, Matsuyama and Moji [13] reported that bleeding after delivery is considered natural in Nepal and is thus not recognized as a danger sign. Similar findings in Bangladesh showed that there was significant disagreement among women, skilled birth attendants, and traditional birth attendants about what constitutes excessive bleeding after birth and what quantity of blood is life-threatening [14]. Thaddeus et al. [15] described a “mismatch” between local perceptions of normal versus potentially dangerous postpartum bleeding, which can give rise to reduced urgency in seeking emergency care.

#### Newborn

Recognition of neonatal illness can be difficult for family members and health professionals due to the lack of specific signs [16–18]. Several newborn signs are not well recognized as signaling danger, such as small size [17, 19], fast breathing [20, 21], yellowing of skin and eyes, abdominal distension, and poor sucking and feeding [20]. The perception of illness severity is also a very important motivator of care seeking; families are more likely to seek care when they perceive the signs to be severe [22–25]. Also, behavioral changes in the neonate may trigger the care-seeking process such as change in feeding behavior [20, 24], sleeping behavior [20], exhibiting grunting [23], lethargy, lack of movement, or a weak or abnormal cry [24].

### Delay 2: seeking care

There is significant overlap regarding barriers to care seeking for maternal and newborn illness. Financial constraints represent the most significant barrier to seeking care [12, 26–31]. Another common barrier is the preference for traditional providers or family members over skilled or professional care [7, 20, 26, 32–36]. If families perceive the cause of illness to be spiritual in nature, they are more likely to seek care from a religious or traditional healer before seeking skilled care [37, 38]. Additional factors correlating with lower likelihood of care seeking include low level of education [26, 29, 38, 39] and lack of decision-making power among women [26, 28, 36, 39]. Concerns about the capacity of health facilities to diagnose and treat illness may also discourage women and their caretakers from seeking skilled or professional care outside the home [27, 36]. Several studies have shown that disrespect and abuse of women is a deterrent to seeking skilled care [26, 40].

For newborns, there are additional considerations inhibiting or delaying care seeking such as low self-efficacy to influence the clinical course of newborn illness [39]; local concerns about malevolent spirits or harmful effects of medical treatment; postpartum confinement of mother and newborn [41]; influence of local beliefs that perceive certain danger signs like trouble breathing and sunken eyes to be untreatable by modern medicine [42]; and discouragement of care seeking from religious elders [23].

There are several facilitators to seeking skilled care. For example, the use of antenatal care services has been linked with care seeking for maternal complications [10, 28, 43]. Various types of support including the support of community health workers [43] and access to a social network [28] have all been linked with care seeking.

## Methods

**Fig. 1:**
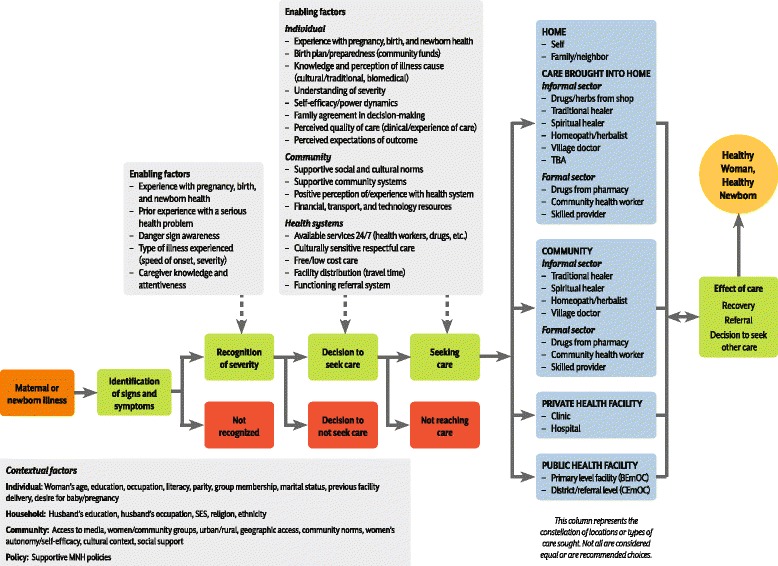
Conceptual framework for recognition and care-seeking for maternal and newborn illness

To guide the research, a conceptual framework was developed, adapting the Three Delays Model (Fig. 1), the MotherCare project framework, and the Pathway to Survival (http://pdf.usaid.gov/pdf_docs/Pnabz644.pdf). Based on the literature review and the MotherCare Framework, the first delay was modified to include not only identification of signs/symptoms of illness but the recognition of severity. In addition, the second delay was modified to better reflect decision-making processes that lead to different care-seeking actions. Finally, we included a variety of care-seeking patterns, including bringing care to the home (e.g., skilled providers and medicines, as well as traditional/spiritual remedies). This research study assessed each of these constructs among women and newborns who survived and those who died in seven different country study sites.

The United States Agency for International Development (USAID) Translating Research into Action (TRAction) project staff and USAID colleagues developed a call for applications. Through this competitive process, six applications were selected to participate under TRAction (Ethiopia, India, Indonesia, Nigeria, Tanzania (study not presented in this supplement), and Uganda), and one additional study site (Nepal) later joined with funding through the USAID-supported Health Research Challenge for Impact project. Each study site had an ongoing program to improve maternal and newborn survival, and this protocol was nested into the ongoing program/research.

A protocol development workshop was conducted in February 2014 in Geneva, Switzerland, with each of the country teams (except for Nepal) and other technical experts. The groups worked together to refine the conceptual framework and develop common research questions, methods, and tools, which together constituted a common protocol. This protocol was adapted for each site, as needed, retaining the main research questions and using the same tools and methods.

The following research questions were addressed:

1. What was the process around recognition of and decision-making for seeking treatment/care among the families of women who experienced post-partum hemorrhage or maternal death regardless of birth location within the last 6 months?
2. What was the sequence of actions for seeking treatment/care among these families?
3. What was the process around recognition of and decision-making for seeking treatment/care among families of newborns who experienced an illness compatible with sepsis or died within the first 28 days of life regardless of birth location within the last 6 months?
4. What was the sequence of actions for seeking treatment/care among the families of these newborns?

### Study methods

This study utilized mixed-methods, including event narratives, in-depth interviews, focus group discussions, rapid facility assessments, and secondary analyses of existing program data. A total of seven sites participated in this multi-country study. Table 1 describes each study site.

**Table 1:** Description of study sites

Country	Partner	Existing project setting	Study site	Population
Ethiopia	Emory University	MaNHEP	Amhara and Oromiya regions; 6 districts	350,000
Uganda	Makerere University	EQUIP	Busoga Region; Mayuge and Namaingo districts	693,000
Tanzania	Ifakara Health Institute	EQUIP	Mtwara Region; Tandahimba and Newala districts	433,006
India	Population Council	Rajiv Ghandi Mahila Vikas Pariyojana (RGMVP)	Uttar Pradesh state; Amethi and Raibereli districts	2 million
Indonesia	World Vision		Papua Province, Jayawijaya District	431,338
Nigeria	JPAL	JPAL study	Jigawa state; 96 communities	290,000
Nepal	Johns Hopkins Bloomberg School of Public Health	Nepal Nutrition Intervention Project, Sarlahi (NNIPS)	Sarlahi district; 34 village development committees	300,000

#### Event narratives

Women who had survived reported post-partum hemorrhage were asked to identify two to three other individuals who were present at the illness event (such as birth attendant, female family members, neighbors). In the case of maternal deaths, the family was contacted and asked to identify two or three other persons who were present at the illness event as described above. For newborn illnesses and deaths, the families were contacted and asked to identify two to three persons who were present over the duration of the illness event (such as the mother, the father, female family members, caregivers). A trained interviewer conducted group interviews with the identified participants for each case type.

A separate instrument was developed for women and newborns cases. Both contained open- and closed-ended questions designed to elicit the perceived onset and sequence of signs and symptoms, emotional and cognitive reactions, behavioral responses, and the process of decision-making for identified signs and symptoms as experienced by the informants, *as these unfolded over time*. The instrument included a structured timeline adapted from previous research studies to facilitate the recording of the event [29]. The instrument also permitted the textual recording of responses by specific group members that detail the circumstances surrounding the event and any factors associated with the identification of and response to the event. Included was a record of decision-making processes, preferred types of treatment/care, and the perceived quality of care and economic or logistical barriers to care seeking. Informants’ demographic and social characteristics were also captured. In addition to the group interviews for the event narratives, some study sites also conducted in-depth interviews and focus group discussions to further explore key research questions.

Instruments were translated into the local language and reviewed for accuracy by the principal investigator, research manager, and data collectors in each study site. Pairs consisting of a trained interviewer and a note taker conducted and audiotaped the face-to-face group interviews in the local language in each setting. Each 60–90-min interview was conducted at a time and place that was convenient for the informants. During the interview, and at its completion, the interviewers reviewed the entire sequence of perceived signs and symptoms, emotional and cognitive reactions, and actions taken in response to the event. By doing so, the interviewers stimulated recall and verified and clarified ambiguities in the event history.

A training of study team principal investigators/research managers was conducted in Addis Ababa, Ethiopia, with representatives from each study team, except for Nepal. This training included detailed description of the study methods, study tools, and pilot testing in the Ethiopia context. The training was replicated in each study site by the principal investigator/research manager, using the materials and tools from the Addis Ababa workshop.

#### In-depth interviews and focus group discussions

In addition to the event narratives, some study sites conducted in-depth interviews (IDI) with key informants (e.g., community health workers, husbands, community leaders) as well as focus group discussions (FGD) to answer site-specific questions. These IDIs and FGDs were used to supplement information from the event narratives.

#### Facility assessment

All sites conducted a rapid facility assessment once during the data collection period, based on a structured questionnaire to assess availability of key maternal and newborn services, health providers, and essential equipment and supplies.

Data collector trainings were conducted with technical support from TRAction and regional social science experts to ensure consistency across the sites and to make adjustments as needed. All data collection tools were extensively pilot-tested, with cross-country consistency and quality control achieved by having one or more common resource persons at two or more data collection training and field testing sessions.

### Sample size

The common protocol required, at a minimum, five cases of each type (e.g., perceived postpartum hemorrhage, maternal death, newborn illness, and newborn death) for each study site. However, due to difficulty identifying maternal deaths (and the small population of some study sites), the numbers of maternal deaths varied between three to five cases per study site. Several research teams were interested in looking at differences between intervention and control areas, or urban and rural areas, and, as a result, increased the sample size to ensure representation of different factors. Table 2 provides a summary of the sample for all sites.

**Table 2:** Sample size across sites and by types of cases

Country	Maternal illness	Maternal death	Newborn illness	Newborn death	Additional IDIs/FDGs	Facilities assessed
Ethiopia	17	5	16	13	0	21
Uganda	16	8	16	8	6	4
Tanzania	16	8	16	8	5	10
Nigeria	10	10	10	10	18	24
India	10	6	10	6	20	11
Indonesia	5	2	5	4	5	5
Nepal	10	6	10	6	10	24
Total	84	45	83	55	64	99

### Case selection

Based on the common protocol, there were standard inclusion and exclusion criteria for each case as follows:

Inclusion criteria:

Maternal

1. Reported excessive blood loss at the last birth in the last 6 months
2. Reported maternal death in the last 6 months

Newborn

3. Reported newborn illness in the last 6 months (some cases within 0–7 days and some cases 8–28 days)
4. Reported newborn death in the last 6 months (some cases within 0–7 days and some cases 8–28 days)

Exclusion criteria:

1. Newborns born in a facility who developed illness and/or died prior to discharge
2. Women who gave birth in a facility and developed postpartum hemorrhage and/or died prior to discharge
3. Any case that occurred *more* than 6 months prior to the interview

Procedures for case selection varied depending on the study site and ongoing research or program. Some sites relied on reports from community health workers and community-based informants, while other sites utilized facility-based records and discussions with health providers. One site in India conducted a household survey, which included questions to identify both illness and death cases.

Once cases were identified, researchers used a standard screening form to ensure they met the inclusion criteria. If the case met the inclusion criteria, the respondent was asked to identify two to three other respondents who participated in the event (e.g., illness or final illness that led to death). If consent was obtained, a group interview was scheduled in a private location. In some instances—for example, in Nepal—it was difficult or not culturally appropriate to conduct a group interview; in those cases, separate in-depth interviews were conducted as needed.

#### Ethical approval

Informed consent was obtained from all respondents. Ethical approval was obtained for each research study, based on local requirements (for more details, please see each individual paper).

#### Analysis

Event narratives were transcribed and translated into English by the data collectors or hired translators. Transcripts were coded for content analysis using a standard codebook, developed a priori. The analysis included coding within and across cases, identifying recognition categories and broad themes, and focusing on recognition of illness, decision-making, and patterns of care seeking, outcomes, and the contextual factors influencing recognition of and response to the illness. Examples of the contextual factors include, apart from the types of content described above, the social affiliations among the focal person’s personal network and characteristics of the event setting. To provide depth, and to illustrate the framework, we also coded narrative quality, noting the presence, content, and resolution of contradictory statements among informants in the group. Each study team used a qualitative analysis software to facilitate analysis including Nvivo, Atlas Ti, and Dedoose (see individual papers for additional details). Once data were coded, each site developed matrices for each case along the research domains (recognition, decision-making, and care seeking), including key quotations. These matrices were standard across sites and facilitated summarizing the findings.

#### Quality assurance

Quality assurance was supported throughout the study through numerous activities, including a joint protocol workshop, cross-participation in data collection trainings, field support during data collection by regional consultants and TRAction staff, communications between study teams about challenges and lessons learned, and cross-site collaboration and TRAction-provided support for analysis and writing.

Each study site was supported by a regional consultant with expertise in social science research and qualitative methods and was fluent in the local language. These consultants worked closely with the study investigators to ensure consistency across study sites, as well as quality within each study site. The consultants participated in the training of data collectors, pilot testing, review of transcripts and translations, re-training as necessary, and support with data analysis and report writing for each study team. For each interview, the data collector completed a standard debriefing template which outlined the key highlights on the interview. Each day, these debriefing templates were reviewed by the study supervisor to ensure quality data collection. In all sites, a sample of transcripts were reviewed for accuracy, including review of audio recordings and translations.

TRAction conducted data analysis workshops in each study site, except for Nepal, to promote consistency in analytic methods across sites. This training included introduction to qualitative analysis methods, the standard codebook, discussion about the matrices, key themes, how to identify quotations, and how to utilize the timelines to facilitate synthesis of findings. These workshops were facilitated by TRAction, USAID, and the regional consultants. A data analysis workshop with all study teams was conducted in August 2015 in Delhi, India, to discuss preliminary findings from each of the study sites and update the conceptual model and further analyses. In this workshop, standard visualizations for care-seeking patterns of timing of care seeking were developed and adopted for each study site.

A writing workshop was conducted in Washington, DC, in April 2016 to finalize the results and papers.

## Discussion

The primary objective of the multi-site study was to explore research questions in depth and synthesize general similarities and differences in the aggregate across all cases—between maternal and newborn and, where possible, between deaths and illness. The setting-specific findings are invaluable in characterizing local illness recognition and care-seeking patterns and, however, cannot be used to generalize to an entire region or country. Still, the strength of the study lies in the ability to pool results across sites to extract patterns that may apply across countries and cultural contexts, as well as differences. Another strength lies in the speed and cost with which this type of study can be undertaken. With experienced interviewers, the depth and nuance of the information collected can be extremely valuable to understanding the context in which a program is being implemented and can inform program implementation in almost real time.

The methods used in this study are not without weaknesses. Because of the labor-intensive nature of the event narrative methodology combined with the relatively rare event of mortality, especially maternal mortality, the sample size per study site is relatively small. Another inherent methodological limitation is reliance on self-report and recall of events and the associated feelings, beliefs, circumstances, and reasoning processes that were at play at the time of the illness event. Given the myriad details that respondents are asked to remember and re-tell, there may be issues with recall. In some instances, the exact timing of events was difficult to document as well as the precise sequence of events. To minimize this bias, we restricted the maximum recall period to 6 months which was shorter in some sites. Additionally, the methodology allows for respondents present during the illness event to participate in the interview to mitigate the loss in information that might result from relying on just one respondent’s account.

Finally, this study focused on maternal illness of perceived postpartum hemorrhage. The study team decided to focus on postpartum hemorrhage because it is the leading cause of maternal mortality worldwide [44] and because of the limited sample size and overall study objectives. Other common maternal illnesses associated with conditions such as pre-eclampsia/eclampsia were not included, due to the different nature of type and onset of symptoms. We anticipate that recognition and care-seeking actions could be very different for conditions other than PPH. It would be useful to replicate this study for other maternal illness. Similarly, because of the difficulty in differentiating between signs and symptoms of newborn illness, the study team included all cases that reported any illness or death. As a result, there is a possibility that a newborn was not actually experiencing a medically defined severe illness but the analysis of the steps taken to address the perceived illness was valuable nonetheless.

## Conclusion

Maternal and newborn mortality remain a significant public health concern in low- and middle-income countries. To achieve ambitious SDG targets, acceleration in the use of quality and respectful maternal and newborn care is essential, as well as focusing on the continuum of care. This study provides rigorous evidence on how women and families recognize illness and potential life-threatening conditions and how decisions are made and describes care-seeking patterns. By using a common methodology and tools, findings were site-specific but also allow for comparison across contexts. The process was facilitated through technical assistance at multiple time points for capacity building, quality assurance, and consistency across sites.
